# Effects of Deoxyschisandrin on Visceral Sensitivity of Mice with Inflammatory Bowel Disease

**DOI:** 10.1155/2019/2986097

**Published:** 2019-12-04

**Authors:** Zhili Xu, Mingbo Zhang, Deqiang Dou, Tingguo Kang, Feng Li

**Affiliations:** ^1^College of Pharmacy, Liaoning University of Traditional Chinese Medicine, Shenyang, Liaoning 116600, China; ^2^Department of Interventional Therapy, First Affiliated Hospital of Dalian Medical University, Dalian, Liaoning 116001, China

## Abstract

The aims of this study were to build an IBD mouse model and further to observe the effects of deoxyschisandrin on IBD and visceral sensitivity and to evaluate the relevance of brain-derived neurotrophic factor (BDNF) to intestinal hypersensitivity of IBD mice. The results showed that deoxyschisandrin could depress the contraction of isolated smooth muscle, modulate gastrointestinal function, and efficiently decrease the disease activity index (DAI) of IBD mice, which proved that deoxyschisandrin had antidiarrheal effects on the animals. In the colorectal distention (CRD) experiment, visceral sensibility was increased in the model group. However, abdominal withdrawal reflex (AWR) scores were decreased after deoxyschisandrin intervention, indicating that deoxyschisandrin could reduce the visceral hypersensitivity of IBD mice. Both IHC observation and western blotting analysis showed that BDNF protein expression increased evidently in colon of IBD mice. After the intervention of deoxyschisandrin, colon mucosa BDNF protein expression in IBD mice decreased, indicating that deoxyschisandrin could decrease mouse intestinal sensitivity by reducing colon mucosa BDNF expression. In conclusion, deoxyschisandrin possessed antidiarrheal effects and visceral hypersensitivity inhibitory effects in the mice with IBD induced by TNBS, which was related to the reduction in BDNF expression in the colon.

## 1. Introduction

Inflammatory bowel disease (IBD), including Crohn's disease and ulcerative colitis, is chronic recurrent gastrointestinal inflammation, which is believed to occur in individuals with genetic susceptibility due to the exposure to unknown environmental and microbial agents [1]. Ulcerative colitis is characterized by continuous inflammation of the colonic lamina propria, followed by damage and destruction of the mucosal barrier. Crohn's disease, by contrast, is characterized by transmural inflammation of any part of the gastrointestinal tract but most ordinarily the area adjacent to the ileocecal valve. IBD patients usually show abdominal discomfort and pain and other clinical symptoms [2, 3]. In human study, IBD patients had visceral hypersensitivity even if their disease was in static condition [4–7], and experimental IBD animals showed increased intestinal sensitivity [8]. In fact, there was ample evidence that colitis could cause motility changes and visceral allergies in various models. Therefore, visceral sensitivity increasing might have a pathophysiological relationship with the generation of symptoms in IBD.

**Figure 1:**
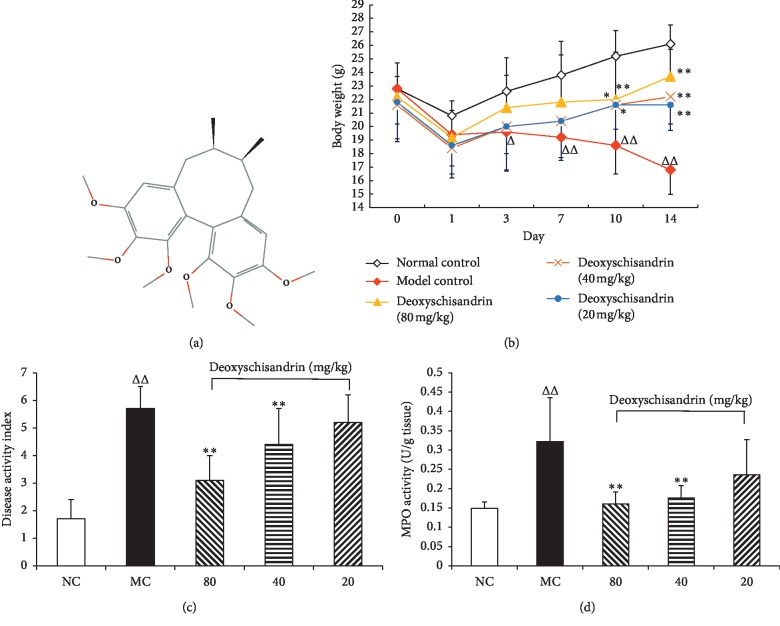
Effects of deoxyschisandrin on the body weight, DAI score, and MPO activity of IBD mice. (a) Representative chemical structure of deoxyschisandrin. (b)–(d) Changes in body weight, DAI score, and MPO activity, respectively. ^*△*^*P* < 0.05 and ^*△△*^*P* < 0.01 vs. normal control; ^*∗*^*P* < 0.05 and ^*∗∗*^*P* < 0.01 vs. model control.

Deoxyschisandrin (Figure 1(a)) is a bioactive lignin compound with potential neuroprotective effects, isolated from the fructification of *Schisandra chinensis* (Turcz.) Baill, which has been used extensively to treat spontaneous sweating, chronic asthma, insomnia, and amnesia as a traditional Chinese medicine for centuries [9]. The most important active ingredients of *Schisandra chinensis* (Trucz.) Baill are lignans with dibenzocyclooctadiene skeletons, such as schisandrol A, schisandrol B, deoxyschisandrin, and schisandrin B [10]. Recent studies have reported that deoxyschisandrin has useful pharmacological actions, for instance, anti-inflammation, antioxidation, antitumor, and hepatoprotection activities [11–14]. However, its influence on intestinal sensitivity and relevant mechanisms in IBD has been rarely reported.

Brain-derived neurotrophic factor (BDNF) is broadly spread in the urinary bladder, lung, colon, skin, and nervous system [15]. BDNF possesses significant effects on differentiation, growth, and damage restoring and could maintain the normal function of sensory nerve [16, 17]. The increased level of BDNF may result in various abnormal feelings related to pains such as chronic pain, inflammatory pain, visceral pain, and high sensitivity [18].

The aims of this study were to build an IBD mouse model and further to examine the effects of deoxyschisandrin on IBD and visceral sensitivity and the relationship between BDNF and intestinal hypersensitivity of IBD mice. Besides, the effects of deoxyschisandrin on the contractibility of isolated jejunal segment (IJS) rats were also observed.

## 2. Methods

### 2.1. Animals and Reagents

The experiments were implemented based on the regulations of animal care and were authorized by the animal ethics committee of Liaoning University of Traditional Chinese Medicine, credential No. SYXK (Liao) 2013-0009. The animal feeding facility was in accordance with the national standard of China (Laboratory Animal-Requirements of Environment and Housing Facilities) (GB 14925-2001).

Fifty male Kunming strain mice, 18∼22 g, and 40 male Sprague-Dawley (SD) rats, 180∼220 g, were acquired from Liaoning Changsheng Biotechnology Co., Ltd., qualified by approval No. SCXK (Liao) 2015-0001. Mice were kept five in one cage in a room with constant temperature (12 h/12 h light/dark, free diet) for 7 days before experimentation.

Deoxyschisandrin (>98% pure, free of endotoxin) was purchased from Nanjing DASF Biotechnology Co., Ltd. (Lot No. W-005-151221). Trinitrobenzene sulfonic acid (TNBS) was obtained from Sigma (Lot No. SLBP0899V). Unless otherwise indicated, chemical agents were obtained from Sigma (USA). Other used chemical agents were of analytical grade available on the market. The concentrations of components (in mmol/L) in Krebs's solution were as follows: sodium dehydrogenate phosphate, 1.8; sodium chloride, 114.0; potassium chloride, 4.7; magnesium chloride, 1.2; calcium chloride, 2.5; glucose, 11.5; and sodium bicarbonate, 18.0 (pH 7.4).

### 2.2. Measurement of the Motility of the Isolated Jejunal Segment (IJS) in Rats

Abdominal rupture in rats was performed after ether anesthesia, and then the small intestines were made as reported earlier [19, 20]. The IJS was sheared in about 2 cm of tubes for detecting the influence of deoxyschisandrin on the contractility of IJS smooth muscle in rats. Mucosa and submucosa were removed gently with fine tweezers, and IJS was suspended in 20 ml chambers, respectively, prefilled with 37°C Kreb's solution and supplied with 95% O_2_ and 5% CO_2_. Contracting in response was immediately determined through BL-420F physiological recording system, Chengdu Techman Software Co. LTD., China. Contractile amplitude of smooth muscle recorded was expressed as the relative value, and the average recording time of the contractile curve should be over 5 min. The contractile amplitude of normal control (Krebs' solution without deoxyschisandrin) was assigned a value of 100%. The average amplitude of contraction was counted from six independent assays.

### 2.3. Animal Model of IBD Construction and Treatment

The mice were randomly divided into five groups, 10 mice in each group, namely, normal control group (NC), model control group (MC), and high, middle, and low dose of deoxyschisandrin groups (80, 40, and 20 mg/kg), respectively.

IBD was induced by TNBS in accordance with the method reported previously with some modifications [21]. Briefly, mice were fasted overnight and moderately anesthetized with ether. For inducing colitis, 5 mg TNBS was uniformly soluble in 0.2 ml of 50% ethanol. Then, a 16-gauge lavage needle was inserted into the anus advanced to 4∼5 cm proximal, and the mixed solution was slowly injected into the descending colon. In order to ensure the retention of TNBS within the entire colon and cecum, the mouse's tail was held in a perpendicular position for 30 seconds after enemata. Mice from the noncolitis of the NC group were given the enema with 0.2 ml of 50% ethanol alone by the same operation. After 24 h, NC group and MC group were given saline intragastrically, and high, middle, and low dose of treatment groups were given orally deoxyschisandrin (80, 40, and 20 mg/kg) once daily, respectively. The mice in each group were administered continuously for 14 days.

### 2.4. Evaluation of IBD in Mice

The evaluation of the IBD severity was investigated by recording the body weight of mice, disease activity index (DAI) evaluation, and myeloperoxidase (MPO) activity. The mice were surveyed every day for variation in body weight, mentality, activity, hair luster, anorexia, and cacation (e.g., whether bloody stool, stool pattern, and defecate frequency). Each mouse was scored for DAI given by stool performance score plus blood stool score [22, 23]. The benzidine method was used to detect the degree of fecal occult blood.

MPO activity was measured using the MPO assay kit (Nanjing Jiancheng Bioengineering Institute, Lot No. 17020220). The whole experiment was performed according to the MPO assay kit's illustration and was repeated 3 times independently.

### 2.5. Abdominal Withdrawal Reflex Experiments: Intestinal Tract Sensitivity Determination by Colorectal Distention (CRD) Test Grades of Abdominal Withdrawal Reflex (AWR) [24, 25]

Score standards are as follows: 0, the mice had stable emotion when given CRD stimulation but had no behavioral response to distention; 1, the mice were in emotional instability, twisting the head; 2, the back and abdominal muscles of mice were contracted lightly but their bellies were not lifted; 3, the back and abdominal muscles of mice were contracted strongly and their bellies were lifted off the floor; 4, the belly muscles of mice were contracted more strongly, their backs were arched, and their bellies, pelvis, and perineum were lifted off the floor.

After 24 h fasting but free access to water, visceral sensitivity was determined by behavioral reactions to CRD. On the day of testing, one hour after the administration, the mice were lightly sedated with ether for 8F catheter insertion with liquid paraffin oil, leaving 1 cm between the gasbag-end and anus, and secured by taping the attached tubing to the mouse tail. Then, mice were planted inside a restraint device. They were allowed to recover 30 min fully from the anesthesia and throughout the duration of colorectal distention testing. Warm saline (37∼38°C) was injected into the gasbag of catheter in 20 min later; the volume was gradually increased by 0.02, 0.04, 0.08, and 0.16 ml (each volume was lasted for 20 s and then had a break for 4 min). Two observers conducted AWR grading separately; if AWR grade was 4, no more volume experiment would be done. There was a 30-minute break between the two CRDs; AWR grading was repeated twice on each mouse for an average grade.

### 2.6. Staining Process of Hematoxylin and Eosin (H&E)

The histopathological changes of colonic tissues were observed under light microscope. The mice were executed, and one 1 cm colon (anus front 5 cm) was anatomized and cleaned with 4% formaldehyde. For the sake of histological evaluation, the colonic samples were fixed with 10% formaldehyde, routinely dehydrated and embedded, cut into 5 *μ*m thick sections, and then stained with H&E. The histological damage was assessed according to previously reported criteria [3, 26].

### 2.7. ELISA Assay of BDNF Expression

Enzyme-linked immunosorbent assay (ELISA) kit (Nanjing Jiancheng Bioengineering Institute, China, Lot No. 20170103) was used to detect the expression of BDNF in colon of mice. The operation procedures were executed according to the kit illustrations. The OD value was detected at 450 nm and the concentration was counted on the basis of the standard curve.

### 2.8. Immunohistochemistry (IHC) of BDNF Expression

The routine operations of IHC are executed following the guide of kit instructions [27]. The brief description is as follows. The sections of colonic tissue were deparaffinized with xylene, hydrated with gradient ethyl alcohol, incubated with 0.1% Triton X-100, and then washed three times with PBS. The sections were blocked by 5% bovine serum albumin and 10% sheep serum successively. The sections were incubated separately with the first antibody of BDNF (No. ab108319 abcam (Hong Kong) Ltd., UK) and second antibody, developed by DAB, microscopically observed, and photographed [27]. The immunohistochemical staining was evaluated semiquantitatively using ImageJ 1.52o version Java 1.8.0_112 (NIH, Bethesda, Maryland, USA) according to the methods of [28, 29].

### 2.9. Western Blot Analysis of BDNF Expression

BDNF expression in colon of IBD mice was observed using western blotting (WB) as reported previously [30]. 80, 40, and 20 mg/kg of deoxyschisandrin were administrated intragastrically to IBD mice daily, respectively, as mentioned above. After 2 weeks of deoxyschisandrin administration, mice were put to execution and colon segments were isolated. Total protein was extracted from the full thickness of colonic wall by the application of the Total Protein Extraction Kit (Nanjing Jiancheng Bioengineering Institute, Lot No. 20170910). Anti-BDNF mAb (1 : 1000 dilution) was incubated overnight at 4°C, followed by incubation with 1 : 1000 diluted HRP-conjugated goat antibody against rabbit IgG and stained with an enhanced DAB chromogenic kit (Nanjing Jiancheng Bioengineering Institute, Lot No. 20171203). Six experiments were performed independently, and the bands were detected and quantified with ImageJ software.

### 2.10. Statistical Analysis Approach

One-way ANOVA employing the SPSS 21 software was carried out to analyze the results of experiments and was shown as means ± standard deviation ($\bar{X}\pm s$). A level of *P* < 0.05 was adopted for statistical significance.

## 3. Results

### 3.1. Contractility of Isolated Intestinal Smooth Muscle in Rats

**Figure 2:**
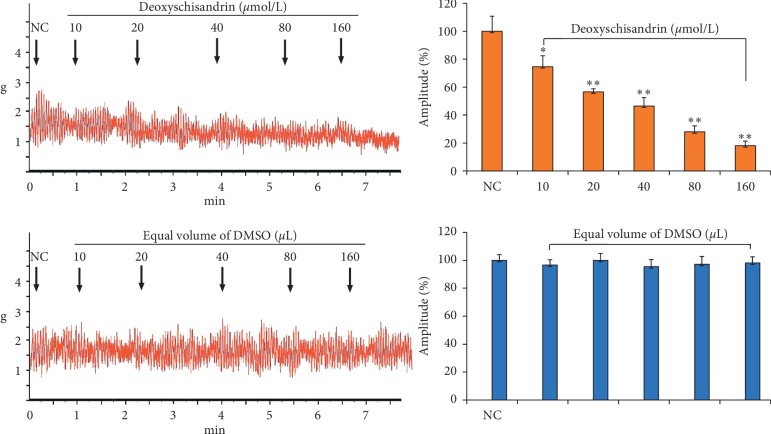
Inhibitory effects of deoxyschisandrin on contractility of isolated intestinal smooth muscle in rats. The contractile amplitude of isolated intestinal smooth muscle preparation before drug treatment is set to a relative value of 100% (normal control, NC). ^*∗*^*P* < 0.05 and ^*∗∗*^*P* < 0.01 vs. NC.

Figure 2 shows that deoxyschisandrin could affect the contractility of IJS incubated in Kreb's solution, which had some dose-response relationship. At the dose of 10∼160 *μ*mol/L, deoxyschisandrin significantly inhibits the contractility of IJS dose dependently (*P* < 0.05, *P* < 0.01).

### 3.2. Body Weight, DAI Score, and MPO Activity of TNBS-Induced Colitis Mice

TNBS modeling was performed from the beginning of the experimental day 0. Changes in body weight of the mice were recorded daily until the end of the experiment. As shown in Figure 1(b), the weight of mice in model group decreased continuously, but the weight loss of mice was inhibited after administration and gradually approached to normal. After administration, the DAI and MPO activities in mice of different groups were determined. As shown in Figures 1(c) and 1(d), treatment with deoxyschisandrin (80, 40 mg/kg) obviously reduced the DAI scores and the level of MPO in comparison with MC group (*P* < 0.01).

### 3.3. Intestinal Tract Sensibility Testing

**Figure 3:**
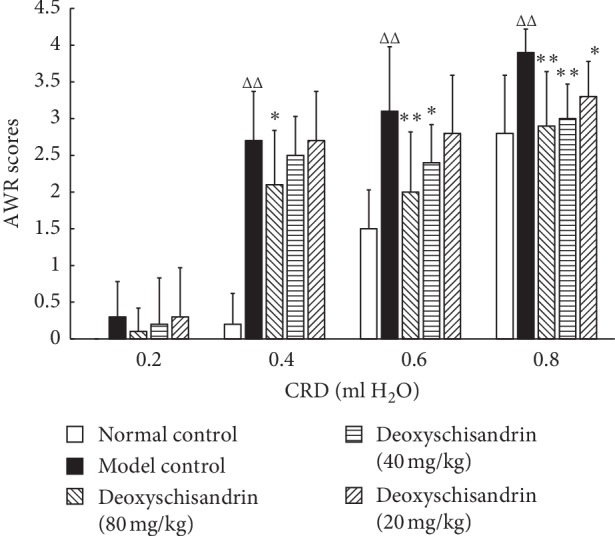
Effects of deoxyschisandrin on AWR scores of IBD mice. AWR scores analyses were performed to investigate the intestinal sensitivity in each group. When the volume of H_2_O was 0.4 ml, ^*△△*^*P* < 0.01 vs. normal control and ^*∗*^*P* < 0.05 vs. model control. When volume of H_2_O was 0.6 ml, ^*△△*^*P* < 0.01 vs. normal control and ^*∗*^*P* < 0.05 and ^*∗∗*^*P* < 0.01 vs. model control. When volume of H_2_O was 0.8 ml, ^*△△*^*P* < 0.01 vs. normal control and ^*∗*^*P* < 0.05 and ^*∗∗*^*P* < 0.01 vs. model control.

When CRD rectal expansion volume was in 0.02, 0.04, 0.08, and 0.16 ml, AWR scores of different groups were indicated in Figure 3. The intestinal sensitivity in MC was much higher than that in NC (*P* < 0.05). After medicine intervention, the intestinal sensitivities in experimental groups were less than those in MC (*P* < 0.05, *P* < 0.01).

### 3.4. Colonic Histomorphology in IBD Mice

**Figure 4:**
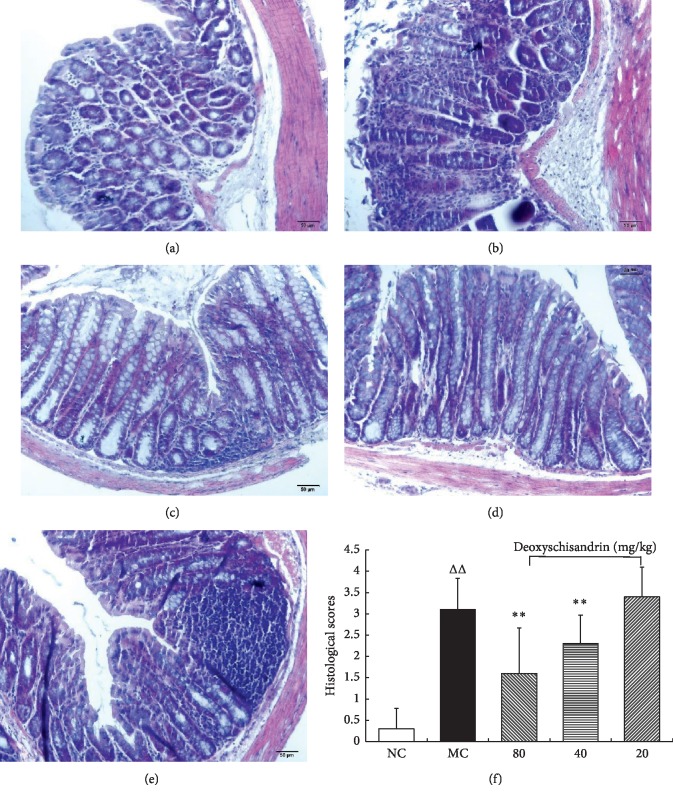
Effects of deoxyschisandrin on the histopathological changes of colons in IBD mice. (a)–(e) H&E staining of colon tissue in IBD mice administrated with deoxyschisandrin. (a) Histological colonic mucosal section of normal control (NC). (b) Histological colonic mucosal sections of model control (MC). (c) Treatment with deoxyschisandrin (80 mg/kg), (d) 40 mg/kg, and (e) 20 mg/kg. (f) The histopathological scores of colons from TNBS-induced colitis mice. ^*△△*^*P* < 0.01 vs. NC; ^*∗*^*P* < 0.05 and ^*∗∗*^*P* < 0.01 vs. MC.

HE staining was performed to testify the efficacy of deoxyschisandrin in relieving the microscopic colonic injury induced by TNBS. The histological inspection showed that, in the colon tissue of the TNBS-induced MC group, the inflammatory cells infiltrated into the mucosa, goblet cells, and epithelium exfoliated, and the architecture of crypts distorted (Figure 4(b)). The deoxyschisandrin treated groups (80 and 40 mg/kg) exhibited the crypt architecture, goblet cells, and epithelium restored successively; and the infiltration of inflammatory cells reduced gradually (Figures 4(c) and 4(d)). The results proved that deoxyschisandrin could markedly improve the histopathological changes of TNBS-induced colitis. Accordingly, the histopathological scores of the deoxyschisandrin administrated groups (80 and 40 mg/kg) showed less value than those of MC group, as shown in Figure 4(f). The score changes confirmed that deoxyschisandrin could alleviate the TNBS-induced inflammation.

### 3.5. BDNF Expression in IBD Mice by IHC

**Figure 5:**
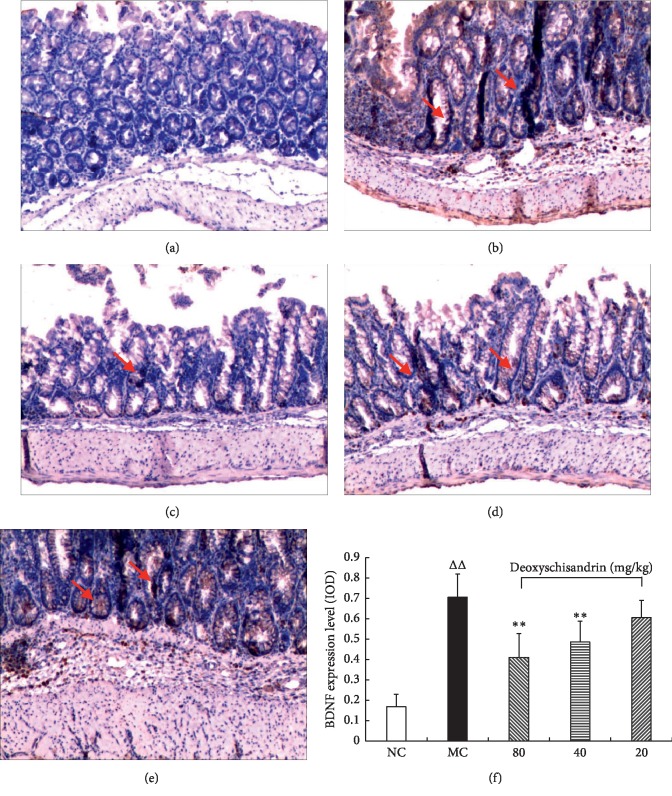
Effects of deoxyschisandrin on the BDNF expression in colon of IBD mice by IHC. (a)–(e) BDNF expression levels were detected by IHC (100×). (a) NC. (b) MC. (c) Deoxyschisandrin group (80 mg/kg). (d) Deoxyschisandrin group (40 mg/kg). (e) Deoxyschisandrin group (20 mg/kg). (f) The semiquantitative evaluation of IHC using ImageJ software. ^*△△*^*P* < 0.01 vs. NC; ^*∗∗*^*P* < 0.01 vs. MC.

Mice colon BDNF IHC results were shown in Figure 5. BDNF is mainly expressed in colon mucosa epithelial cells. In colon, BDNF expression in the MC group was higher than that in the NC group. After drug intervention, BDNF expression decreased evidently in the three experimental groups in comparison with the MC group.

### 3.6. BDNF Expression in IBD Mice by ELISA and WB

**Figure 6:**
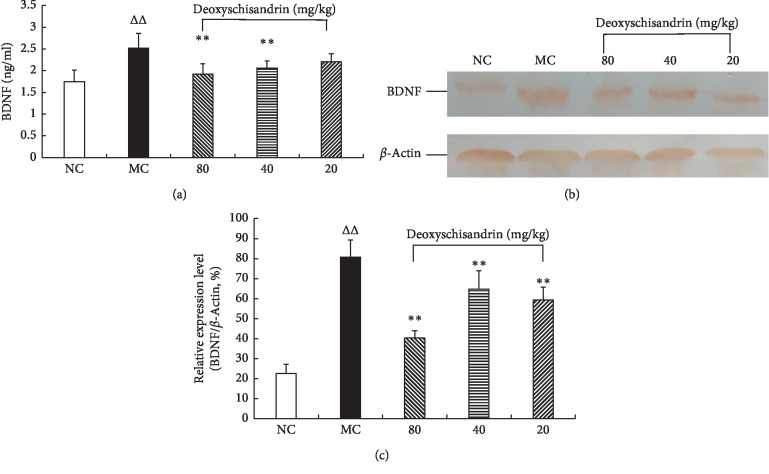
Effects of deoxyschisandrin on BDNF protein expression in colon tissues of IBD mice. (a) ELISA assay was performed to detect the BDNF expression. (b) Electrophoretic strips in WB experiment. (c) Statistical analysis of WB assay. ^*△△*^*P* < 0.01 vs. NC; ^*∗∗*^*P* < 0.01 vs. MC.

Both ELISA and western blotting results indicated that BDNF in mouse colon tissues of the model control increased evidently compared with NC group (*P* < 0.01). After drug intervention, BDNF expression decreased evidently in the three experimental groups in comparison with the MC group (Figure 6).

## 4. Discussion

IBD is a spectrum of chronic, idiopathic, inflammatory intestinal conditions. Clinical symptoms in IBD contain abdominal pain, undernutrition, and diarrhea, often hematochezia. It is generally believed that the incidence of IBD is due to environmental factors interacting with a susceptible host and is closely related to the enteric flora, the intestinal barrier, and immune-related features [31]. According to the theory of traditional Chinese medicine, IBD pertains to the Chinese medical entries of melena, dysentery, hematochezia, diarrhea, intestine stasis, and impeded defecation and can be caused by exogenous evils such as summer-heat and damp, raw, and hot; or by dirty diet and dyspepsia; or by emotional disorder [32].

Abdominal pain is a common manifestation of clinical recurrences of IBD. Additionally, previous studies have shown that the IBD sufferers possess increased rectal sensitivity to rectal dilatation even when in remission [33].

To date, the pharmacological treatment of gastrointestinal diseases including IBD mainly focuses on symptomatic control. However, the management of the mechanism behind abdominal pain, visceral hypersensitivity, is still one of the biggest challenges. Over the past decades, many potential pharmacological targets have been advanced, but regrettably, an effective etiological treatment is still lacking [34].

Our results indicated that deoxyschisandrin could depress the contraction of isolated smooth muscle, exert inhibitory effects on gastrointestinal function, and efficiently decrease the DAI of IBD mice, which proved that deoxyschisandrin had antidiarrheal effect on the animals. In the CRD experiment, visceral pain threshold of IBD mice was decreased and visceral sensibility was increased in MC group. After deoxyschisandrin intervention, in comparison with the MC group, AWR score was decreased, indicating that deoxyschisandrin could reduce the visceral hypersensitivity of IBD mice. All of the results could provide experimental evidence that deoxyschisandrin might be effective for treatment of the patients with the diarrhea of IBD.

Both IHC observation and WB analysis showed that BDNF protein expression increased evidently in colon of IBD mice in model group, suggesting a close relationship between abnormal BDNF increase and intestinal high sensitivity. The results were similar to the patients with functional gastrointestinal disorders, whose BDNF expression in intestine increased significantly and had close relation to the increase of its intestinal tract sensitivity [35]. After the intervention of deoxyschisandrin, colon mucosa BDNF protein expression in IBD mice decreased, indicating that deoxyschisandrin could decrease mouse intestinal sensitivity by reducing colon mucosa BDNF expression, which helped to relieve pain. Certainly, more researches, such as the study on pertinent cell line (e.g., Mode-K, MSIE, or Caco-2), were still needed to reveal the detailed mode of action and the related effective access.

## Data Availability

The data used to support the findings of this study are available from the corresponding author upon request.

## Abbreviations

BDNF: Brain-derived neurotrophic factor
IBD: Inflammatory bowel disease
IJS: Isolated jejunal segment
TNBS: Trinitrobenzene sulfonic acid
DAI: Disease activity index
CRD: Colorectal distention
AWR: Abdominal withdrawal reflex
ELISA: Enzyme-linked immunosorbent assay
MPO: Myeloperoxidase
IHC: Immunohistochemistry.
